# Long noncoding RNA TUG1 regulates degradation of chondrocyte extracellular matrix via miR-320c/MMP-13 axis in osteoarthritis

**DOI:** 10.1515/biol-2021-0037

**Published:** 2021-04-21

**Authors:** Hu Han, Lijuan Liu

**Affiliations:** Department of Rehabilitation, The First People’s Hospital of Jingmen, No. 67 Xiangshan Dadao, Dongbao District, Jingmen 448000, Hubei, China

**Keywords:** osteoarthritis, TUG1, miR-320c, FUT4, IL-1β

## Abstract

Osteoarthritis (OA) is a common chronic joint disease. This study aimed to explore the function of long noncoding RNA taurine-upregulated gene 1 (TUG1) in the progression and initiation of OA. Levels of TUG1, microRNA-320c (miR-320c) and fucosyltransferase 4 (FUT4) were examined via quantitative reverse transcriptase polymerase chain reaction (qRT-PCR). 3-(4,5-Dimethyl-2-thiazolyl)-2,5-diphenyl-2-*H*-tetrazolium bromide and flow cytometry assays were used to detect cell viability and apoptosis, respectively. The expression of relative proteins was measured using Western blot. The interaction between miR-320c and TUG1 or FUT4 was confirmed utilizing dual-luciferase reporter and RNA immunoprecipitation assays. In this study, levels of TUG1 and FUT4 were distinctly upregulated, but miR-320c level significantly decreased in OA tissues and chondrocytes derived from OA tissues as well as in IL-1β-stimulated C28/I2 cells. Mechanically, TUG1 sponged miR-320c and miR-320c targeted FUT4. In addition, TUG1 knockdown accelerated cell proliferation and repressed apoptosis and extracellular matrix (ECM) degradation in IL-1β-induced C28/I2 cells, whereas these effects of TUG1 deletion were rescued by either miR-320c inhibitor or FUT4 upregulation. Meanwhile, TUG1 sponged miR-320c to regulate FUT4 expression in IL-1β-induced C28/I2 cells. Collectively, TUG1 modulated cell proliferation, apoptosis and ECM degradation in IL-1β-induced C28/I2 cells via the miR-320c/FUT4 axis, providing a new insight into the OA treatment.

## 1 Introduction

Osteoarthritis (OA) is an extensively common degenerative joint disease, which is characterized by articular cartilage degradation along with joint inflammation [1]. Chondrocytes are the large proportion of cells in articular cartilage and have fatal roles in cartilage metabolic homeostasis, and dysregulation of chondrocytes is associated with OA pathogenesis [2]. Previous investigations confirmed that chondrocyte viability, apoptosis and breakdown of extracellular matrix (ECM) degradation were considered to be related with the OA development [3,4,5]. However, the efficient treatment methods for OA require further investigation [6]. Therefore, it is significant to study the pathophysiology and regulatory mechanisms of human OA.

Long noncoding RNAs (lncRNAs) and microRNAs (miRNAs) are noncoding RNAs (ncRNAs). lncRNAs, as a class of ncRNAs with longer than 200 nucleotides [7], have been revealed to possess critical regulatory functions in numerous pathological physiologies, such as cell proliferation, apoptosis and tumor formation [8,9,10]. Currently, lncRNA taurine-upregulated gene 1 (TUG1) was verified to be overexpressed in numerous diseases, including OA. For instance, TUG1 regulated the proliferation, apoptosis and migration in OA cells [11]. Moreover, TUG1 boosted chondrocyte ECM degradation through miR-195/matrix metalloproteinase (MMP)-13 axis in OA [12]. These evidence suggested that TUG1 may have vital functions in the progression of OA.

The miRNAs, a type of small ncRNAs, could curb the expression of the target gene via binding to the target mRNAs’ 3′-untranslated region (3′-UTR), consequently, either blocking mRNA translation or striking mRNA degeneration [13]. The miRNAs have been reported to participate in the development of OA [14]. Previous research indicated that miRNAs could regulate cell growth and apoptosis as well as ECM metabolism [15]. Furthermore, aberrant expression of miRNAs was observed in OA cartilage tissues and closely associated with the development of OA [16]. For example, miR-30a accelerated chondrogenesis via regulating DLL4 [17]. Moreover, microRNA-320c (miR-320c) suppressed OA development via regulating the Wnt-signaling pathway [18]. However, the molecular mechanism of miR-320c in OA pathogenesis remains largely unknown.

Herein we detected the expression of TUG1 in OA tissues and chondrocytes isolated from OA tissues as well as interleukin (IL)-1β-induced C28/I2 cells. Moreover, the biological role of TUG1 in the progression of OA was explored in IL-1β-induced C28/I2 cells.

## 2 Materials and methods

### 2.1 Subjects

The OA cartilage samples (*n* = 40) were obtained from OA patients undergoing knee replacement surgery at The First People’s Hospital of Jingmen. These participants did not receive intraarticular steroid injections for nearly 3 months before surgery. Normal cartilages (*n* = 20) were gained from trauma patients without OA or rheumatoid arthritis history. The clinicopathological features of these patients are presented in Table 1.

**Table 1:** Basic information of the patients

Characteristic	Normal (*n* = 20)	OA (*n* = 40)
Age (mean + SD)	49.8 ± 10.4	52.4 ± 9.5
Sex (female/male)	12/8	24/16
Body mass index (mean)	24.6	25.3
OA stage: early/late		15/25


**Informed consent:** Informed consent has been obtained from all individuals included in this study.
**Ethical approval:** The research related to human use has been complied with all the relevant national regulations, institutional policies and in accordance with the tenets of the Helsinki Declaration and has been approved by the Ethics Committee of The First People’s Hospital of Jingmen.

### 2.2 Cell culture and treatment

Chondrocytes from OA or normal articular cartilage were separated using an *in vitro* two-step collagenase digestion method, then maintained in a sterile culture dish and washed thrice with phosphate-buffered saline (PBS; Gibco, Carlsbad, CA, USA). Additionally, complete medium was used to culture the sterile culture dish containing cartilage fragments of size 1 mm × 1 mm × 1 mm. Next the medium was transferred to a 50 mL centrifuge tube and centrifuged for 5 min at 104.67*g* under cold conditions. Then 5 mL of trypsin (Gibco) was added to digest the chondrocytes. According to the cell density, relative volume cell solution was added to the complete cell culture medium in a humidified CO_2_ incubator (5% CO_2_) for subculture. The isolated chondrocytes were cultured in Dulbecco’s modified Eagle’s medium (DMEM; Gibco) containing 10% fetal bovine serum (FBS; Gibco), 100 IU/mL penicillin and 100 mg/mL streptomycin in an incubator at 37°C with 5% CO_2_.

Human cartilage C28/I2 cells were obtained from Cell Bank of the Chinese Academy of Sciences (Shanghai, China). DMEM-containing Ham’s F12 nutrient medium (1:1, DMEM/F12; Thermo Fisher Scientific, Rockford, IL, USA) supplemented with 10% FBS (Gibco) and penicillin (100 IU/mL, Gibco)‒streptomycin (100 mg/mL, Gibco) was used to culture C28/I2 cells. Cells were incubated at 37°C in a humidified incubator with 5% CO_2_. C28/I2 cells treated with 10 ng/mL IL-1β (Sigma, St. Louis, MO, USA) were used to establish OA cell model *in vitro*. Then the IL-1β-induced C28/I2 cells were cultured for 12 h for subsequent assay.

### 2.3 Quantitative reverse-transcriptase-polymerase chain reaction (qRT-PCR)

Total RNA was extracted from OA tissues, chondrocytes and C28/I2 cells using Trizol (Invitrogen, Carlsbad, CA, USA) in accordance with the manufacturer’s introduction. To determine TUG1, miR-320c and fucosyltransferase 4 (FUT4) levels, reverse transcription (RT) and qRT-PCR kits were used. First, RT reactions were carried out utilizing TaqMan microRNA reverse transcription kit (Thermo Fisher Scientific), and cDNAs were collected in a final volume of 20 µL as the template for qPCR. Next TaqMan Universal PCR Master Mix (Thermo Fisher Scientific) was employed to tenderly mix the reaction solutions according to the manuals. Subsequently, all reaction tubes were placed on a 7900 Real-time system (Applied Biosystems, Rockford, IL, USA). Glyceraldehyde-3-phosphate dehydrogenase (GAPDH) or U6 snRNA was applied as the internal references. Relative expression of TUG1, miR-320c and FUT4 was calculated using the 2^−ΔΔCt^ method. The primers are listed as follows: TUG1 (forward, 5′-TCACAAGGCTGCACCAGATT-3′; reverse, 5′-GTCGGTCACAAAATGCATAGAGG-3'); miR-320c (forward, 5′-ACACTCCAGCTGGGAAAAGCTGGGTTGAGA-3′; reverse, 5′-ACACTCCAGCTGGGTCGCCCTC-3′); FUT4 (forward, 5′-CGGACGTCTTTGTGCCTTAT-3′; reverse, 5′-CGAGGAAAAGCAGGTACGAG-3′); GAPDH (forward, 5′-ATTCCATGGCACCGTCAAGGCTGA-3′; reverse, 5′-TTCTCCATGGTGGTGAAGACGCCA-3′); U6 (forward, 5′-ATTGGAACGATACAGAGAAGATT-3′; reverse, 5′-GGAACGCTTCACGAATTTG-3′). The above primers were synthesized by Genepharma (Shanghai, China).

### 2.4 Transient transfection

Small interfering RNA against TUG1 (si-TUG1) and its corresponding negative control (si-NC), miR-320c mimic (miR-320) and its inhibitor (anti-miR-320), as well as the relative control (miR-NC or anti-miR-NC), overexpression sequences of TUG1 and FUT4 (pcDNA-FUT4), empty pcDNA3.1 vector (pcDNA) synthesized by Genepharma were transfected into C28/I2 cells using Lipofectamine™ 3000 (Invitrogen).

### 2.5 3-(4,5-Dimethyl-2-thiazolyl)-2,5-diphenyl-2-*H*-tetrazolium bromide (MTT) assay

For cell proliferation assay, 5,000 C28/I2 cells were seeded onto the 96-well plate. After 12 h incubation, the MTT solution was added into each well and then the cells were cultured for another 4 h. Then the supernatants were replaced with 150 µL of dimethyl sulfoxide (Sigma). Finally, the optical density of the solution was identified via a microplate reader (Thermo Fisher Scientific).

### 2.6 Flow cytometry

Cell apoptosis was measured by using fluorescein isothiocyanate-labeled Annexin V/propidium iodide (Annexin V-FITC/PI) detection kit (Sangon Biotech, Shanghai, China). At 12 h post-transfection, cells were collected and washed with ice-cold PBS. Subsequently, 1× binding buffer was used to resuspend the cells and 5 µL of annexin V and PI was utilized to stain the apoptotic cells in the dark, according to the manufacturer’s protocol. Finally, the proportion of apoptotic cells was counted via a flow cytometry machine (BD Biosciences, San Jose, CA, USA).

### 2.7 Western blot assay

The total proteins from tissue samples, chondrocytes and C28/I2 cells were harvested. The proteins were separated by sodium dodecyl sulfate–polyacrylamide gel electrophoresis (12%) and transferred onto a nitrocellulose membrane (Thermo Fisher Scientific). Then the membranes were blocked by 5% skim milk powder (Sangon Biotech) for 2 h at room temperature. Subsequently, the membranes were incubated with specific primary antibody primary antibodies overnight at 4°C. The primary antibodies included proliferation-relative protein of cyclin D1 (ab16663, 1:150; Abcam, Cambridge, MA, USA), apoptosis protein of cleaved-caspase 3 (cleaved-casp-3; ab2302, 1:500; Abcam), and ECM degradation-relative proteins of MMP-13 (ab39012, 1:4,500; Abcam), type II collagen (ab34712, 1:3,000 collagen II; Abcam), Aggrecan (ab3778, 1:100; Abcam) and FUT4 (ab181461, 1:1,000; Abcam), and GAPDH (ab8245, 1:5,000; Abcam) were the endogenous references. The next day, the uncombined antibodies were washed trice with tris-buffered saline Tween-20 and then the membranes were covered with matched secondary antibody at room temperature for 1 h. After washing, the blots were visualized using enhanced chemiluminescence (ECL) substrates (Millipore, Bedford, MA, USA) and then photographed.

### 2.8 Dual-luciferase reporter assay

The biological prediction software of starBase was employed to identify the binding sites between miR-320c and TUG1 or FUT4. In accordance with obtained results, the common fragments of miR-320c, wild-type (WT-TUG1) or mutant (MUT-TUG1) TUG1, and 3′-UTRs common sequence of wild-type (WT-FUT4) and mutant (MUT-FUT4) of FUT4 were amplified and cloned into cloning sites downstream of pmirGLO (Promega Corporation, Madison, WI, USA). In this assay, WT-TUG1, MUT-TUG1, WT-FUT4 or MUT-FUT4 was co-transfected with miR-con or miR-320c mimic into cells using Lipofectamine™ 3000 (Invitrogen). Relative dual-luciferase activity assay was analyzed based on the introduction provided by Promega dual-luciferase assay kit. pRL-TK vector (Promega) was used to adjust the cell number and transfection efficiency.

### 2.9 RNA immunoprecipitation (RIP) assay

RIP assay was implemented utilizing Magna RIP RNA-binding protein immunoprecipitation kit (Millipore) and AGO2 antibody in accordance with the producer’s descriptions, and immunoglobulin G antibody was used as the control. Cells were transfected with TUG1, miR-320c mimic or FUT4 and cultured for 24 h, and the abundance was evaluated via qRT-PCR.

### 2.10 Statistical analysis

Data from at least three independent assays were processed by SPSS 19.0 software and expressed as mean ± standard deviation. Student’s *t*-test or one-way analysis of variance was performed to analyze the differences in significant group, and *P* < 0.05 was considered statistically remarkable.

## 3 Results

### 3.1 Increase in TUG1 in OA tissues and cell lines

**Figure 1:**
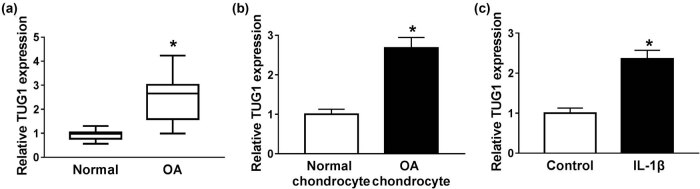
High expression of TUG1 in OA tissues and cell lines. (a) The level of TUG1 was determined via qRT-PCR in OA tissues. (b) qRT-PCR was employed to detect TUG1 level in isolated chondrocytes. (c) Expression of TUG1 in IL-1β-induced C28/I2 cells was identified utilizing qRT-PCR. *P < 0.05.

In order to investigate the biological role of TUG1 in OA development, this study first measured TUG1 level in OA patients, and the result showed that TUG1 expression was efficiently augmented in OA tissues compared with the normal control (Figure 1a). Meanwhile, chondrocytes were isolated from OA and normal tissues. qRT-PCR suggested an increase in TUG1 in isolated OA chondrocytes (Figure 1b). Subsequently, C28/I2 cells and IL-1β were used to establish OA cell model, and the expression of TUG1 was significantly elevated in IL-1β-stimulated C28/I2 cells (Figure 1c). Overall, TUG1 was highly expressed in OA, which may play a critical role in the progress of human OA.

### 3.2 TUG1 deletion promoted cell proliferation and inhibited apoptosis and ECM degradation in IL-1β-induced C28/I2 cells

**Figure 2:**
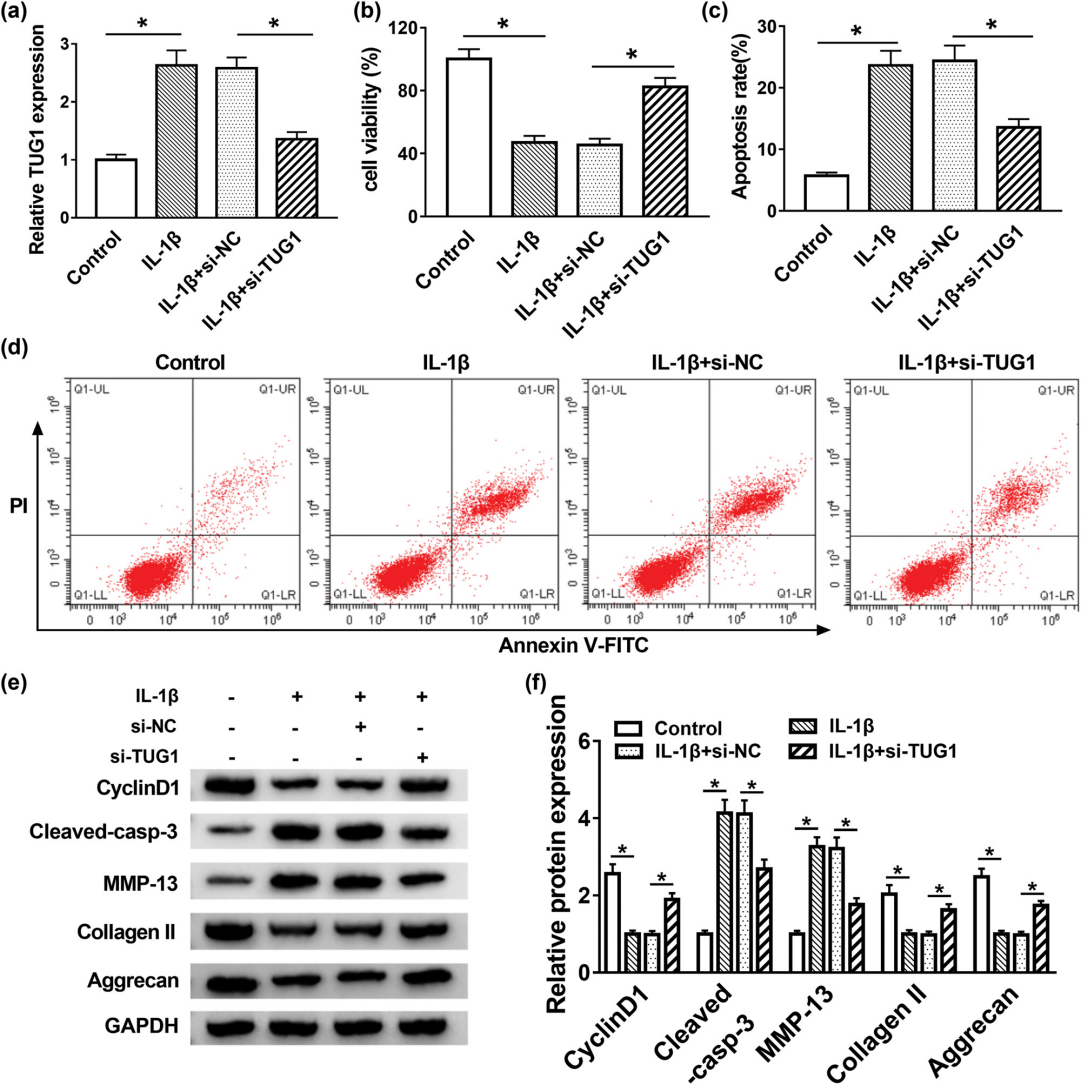
TUG1 deletion promoted cell proliferation and inhibited apoptosis and ECM degradation in IL-1β-induced C28/I2 cells. (a) The knockdown efficiency of si-TUG1 was confirmed via qRT-PCR. (b) MTT assay measured cell proliferation in IL-1β-induced C28/I2 cells. (c and d) Flow cytometry analyzed IL-1β-stimulated C28/I2 cell apoptosis *in vitro*. (e and f) Expression of cyclin D1, cleaved-casp-3, MMP-13, collage II and Aggrecan was measured using Western blot assay. *P < 0.05.

Due to the aberrant expression of TUG1 in OA tissues, the functions of TUG1 were researched *in vitro*. The knockdown efficiency of si-TUG1 was remarkably evident (Figure 2a). The results showed that IL-1β could impede cell proliferation, while this inhibitory effect of IL-1β on cell proliferation was restored by TUG1 knockdown (Figure 2b). Apoptotic cells were also examined by flow cytometry, and the results uncovered that the promotion effect of IL-1β treatment on cell apoptosis was regained via TUG1 silencing (Figure 2c and d). Furthermore, proliferation-related protein of cyclin D1, apoptosis of cleaved-casp-3 and ECM degradation-related proteins of MMP-13, collage II and Aggrecan were measured and quantified in IL-1β-treated C28/I2 cells. The proliferation and apoptosis tendency was in line with the results of MTT and flow cytometry assays, and Western blot results also determined that the acceleration impact of IL-1β on ECM degradation was abrogated by downregulation of TUG1 *in vitro* (Figure 2e and f). In brief, TUG1 knockdown facilitated cell proliferation and relieved apoptosis and ECM degradation in IL-1β-induced C28/I2 cells.

### 3.3 miR-320c was a target gene of TUG1

**Figure 3:**
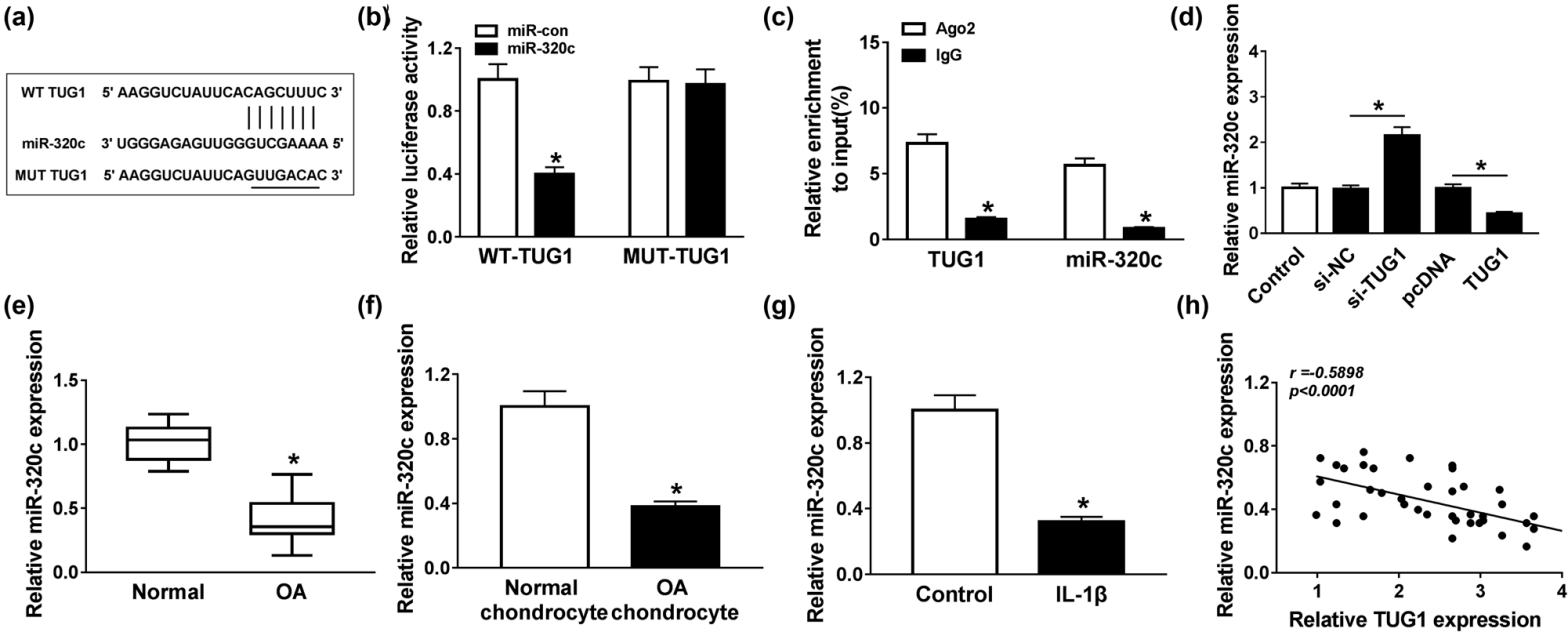
miR-320c was a target gene of TUG1. (a) The interrelation between miR-320c and TUG1 was predicted using starBase software. (b and c) Dual-luciferase reporter and RIP assays verified the interaction between miR-320c and TUG1. (d) Level of miR-320c was examined by qRT-PCR in C28/I2 cells transfected with si-NC, si-TUG1, pcDNA or TUG1, respectively. (e and f) qRT-PCR was utilized to determine the level of miR-320c in OA tissues and isolated chondrocytes. (g) Expression of miR-320c in IL-1β-treated C28/I2 cells was measured by qRT-PCR. (h) The correlation between miR-320c and TUG1 was analyzed by Spearman’s correlation analysis. *P < 0.05.

StarBase software predicted that miR-320c harbored the binding sites for TUG1 (Figure 3a). Dual-luciferase reporter assay indicated that the luciferase activity decreased in the WT-TUG1 group, but not in the MUT-TUG1 group, providing evidence that miR-320c was a target gene of TUG1 (Figure 3b). Furthermore, RIP assay results also concluded that TUG1 directly targeted miR-320c (Figure 3c). Moreover, our data exhibited that TUG1 silencing triggered miR-320c expression, while miR-320c expression was suppressed by the overexpression of TUG1 (Figure 3d). Furthermore, the results demonstrated that the miR-320c level was evidently inhibited in OA cartilage tissues and separated OA chondrocytes (Figure 3e and f). The level of miR-320c was also decreased in IL-1β-treated C28/I2 cells (Figure 3g). Besides, the expression of miR-320c was negatively correlated with the TUG1 level (Figure 3h). All the above data indicated that TUG1 exerted its role in IL-1β-treated C28/I2 cells via downregulating miR-320c.

### 3.4 FUT4 was directly targeted by miR-320c

**Figure 4:**
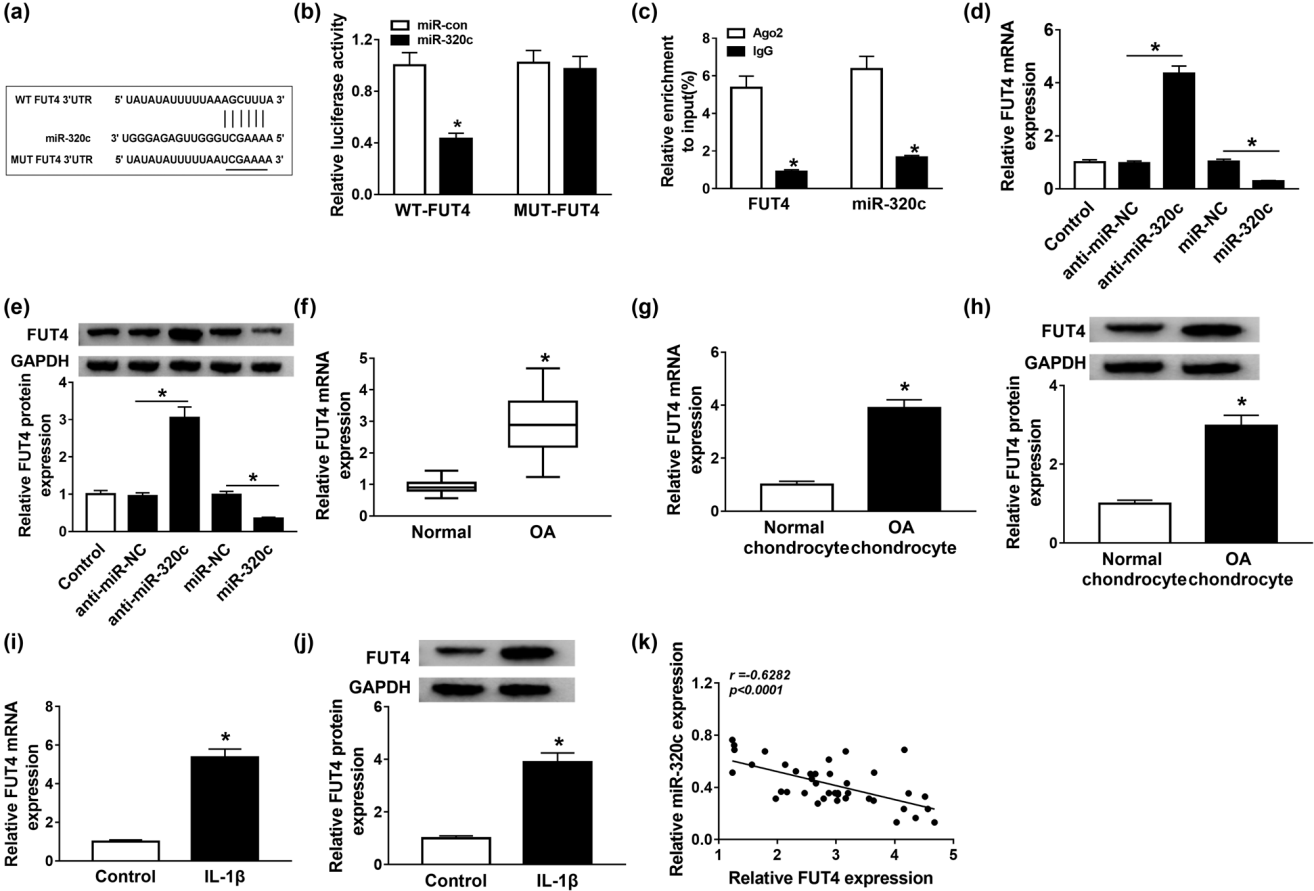
FUT4 was directly targeted by miR-320c. (a) The target relationship between miR-320c and FUT4 was predicted using starBase software. (b and c) The relationship between miR-320c and FUT4 was confirmed via dual-luciferase reporter and RIP assays. (d and e) Expression of FUT4 in C28/I2 cells transfected with miR-320 mimic or its inhibitor was assessed via qRT-PCR and Western blot assays. (f) TUG1 level in OA was analyzed by qRT-PCR. (g–j) The mRNA and protein expression of FUT4 in separated chondrocytes and IL-1β-stimulated C28/I2 cells were examined by qRT-PCR and Western blot assays, respectively. (k) Spearman’s correlation analysis was used to analyze the interrelation between miR-320c and TUG1. *P < 0.05.

StarBase showed that FUT4 was a downstream of miR-320c (Figure 4a). Dual-luciferase reporter assay was used to verify the interaction between miR-320c and FUT4, and the luciferase activity in the WT-FUT4 group was inhibited by miR-320c, while no change was observed in the MUT-FUT4 group (Figure 4b). RIP assay results also demonstrated that FUT4 was targeted by miR-320c (Figure 4c). Furthermore, miR-320c inhibitor apparently boosted FUT4 mRNA level and protein expression, while the effects of miR-320c mimic on FUT4 mRNA level and protein expression were opposite to that of miR-320c inhibitor (Figure 4d and e). Moreover, FUT4 mRNA level specially increased in OA cartilage tissues (Figure 4f). The mRNA level and protein expression of FUT4 were augmented in separated chondrocytes (Figure 4g and h). In addition, the mRNA and protein levels of FUT4 also upregulated in IL-1β-stimulated C28/I2 cells (Figure 4i and j). Our data indicated that FUT4 expression was negatively related to the miR-320c level (Figure 4k). In short, these findings revealed that miR-320c directly targeted FUT4.

### 3.5 FUT4 overexpression reversed the effects of miR-320c on cell proliferation, apoptosis and ECM degradation in IL-1β-treated C28/I2 cells

**Figure 5:**
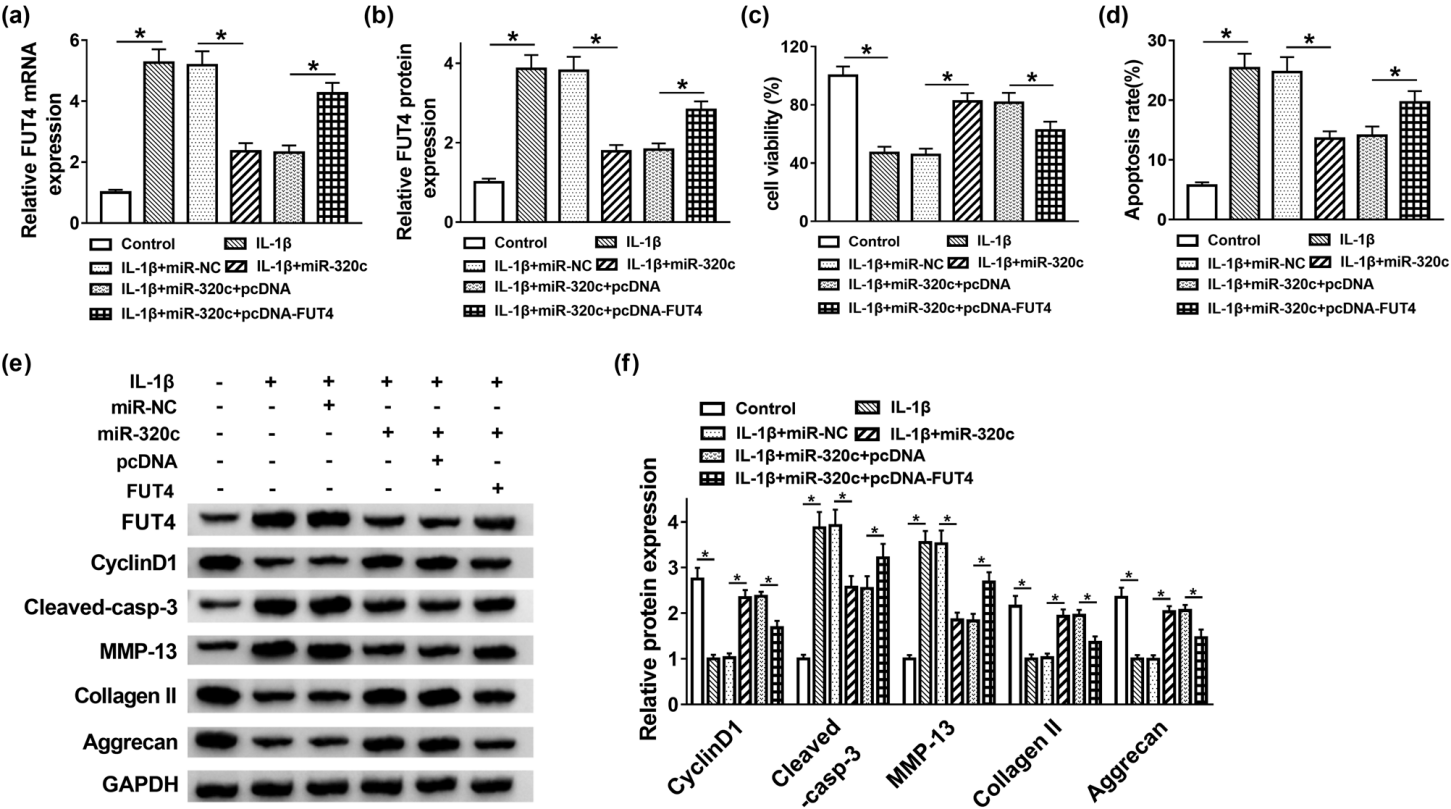
FUT4 overexpression reversed the effects of miR-320c on cell proliferation, apoptosis and degradation in IL-1β-treated C28/I2 cells. (a–f) C28/I2 cells underwent IL-1β stimulation were transfected with miR-NC, miR-320c, miR-320c + pcNDA or miR-320c + FUT4, respectively. (a and b) The mRNA and protein levels of FUT4 were evaluated by qRT-PCR and Western blot assays, respectively. (c) Cell proliferation was measured using MTT assay. (d) Apoptotic cells were calculated via flow cytometry. (e and f) Western blot assay was carried out to detect the protein expression of cyclin D1, cleaved-casp-3, MMP-13, collage II and Aggrecan. *P < 0.05.

To further investigate the regulatory mechanism of miR-320c and FUT4 in the progression of OA, miR-NC, miR-320c, miR-320c + pcDNA or miR-320c + FUT4 was transfected into IL-1β-stimulated C28/I2 cells. The results showed that the inhibiting impacts of miR-320c mimic on the FUT4 mRNA level and protein expression were both rescued by overexpression of FUT4 (Figure 5a and b). Moreover, upregulation of FUT4 relieved the promotion effect of miR-320c mimic on cell proliferation in IL-1β-treated C28/I2 cells (Figure 5c). Cell apoptosis hindered by miR-320c mimic was abrogated by overexpression of FUT4 (Figure 5d). The expression of cyclin D1 and cleaved-casp-3 confirmed the above conclusion of cell proliferation and apoptosis in MTT and flow cytometry assays. In addition, ECM-relative proteins of MMP-13, collage II and Aggrecan were examined by Western blot, and the results indicated that FUT4 upregulation restored the inhibiting effect of miR-320c mimic on degradation of ECM in IL-1β-stimulated C28/I2 cells (Figure 5e and f). These data demonstrated that miR-320c regulated cell proliferation, apoptosis and ECM degradation in IL-1β-treated C28/I2 cells by targeting FUT4.

### 3.6 Effects of TUG1 silencing on cell proliferation, apoptosis and ECM degradation were abrogated by miR-320c inhibitor or FUT4 overexpression in IL-1β-stimulated C28/I2 cells

**Figure 6:**
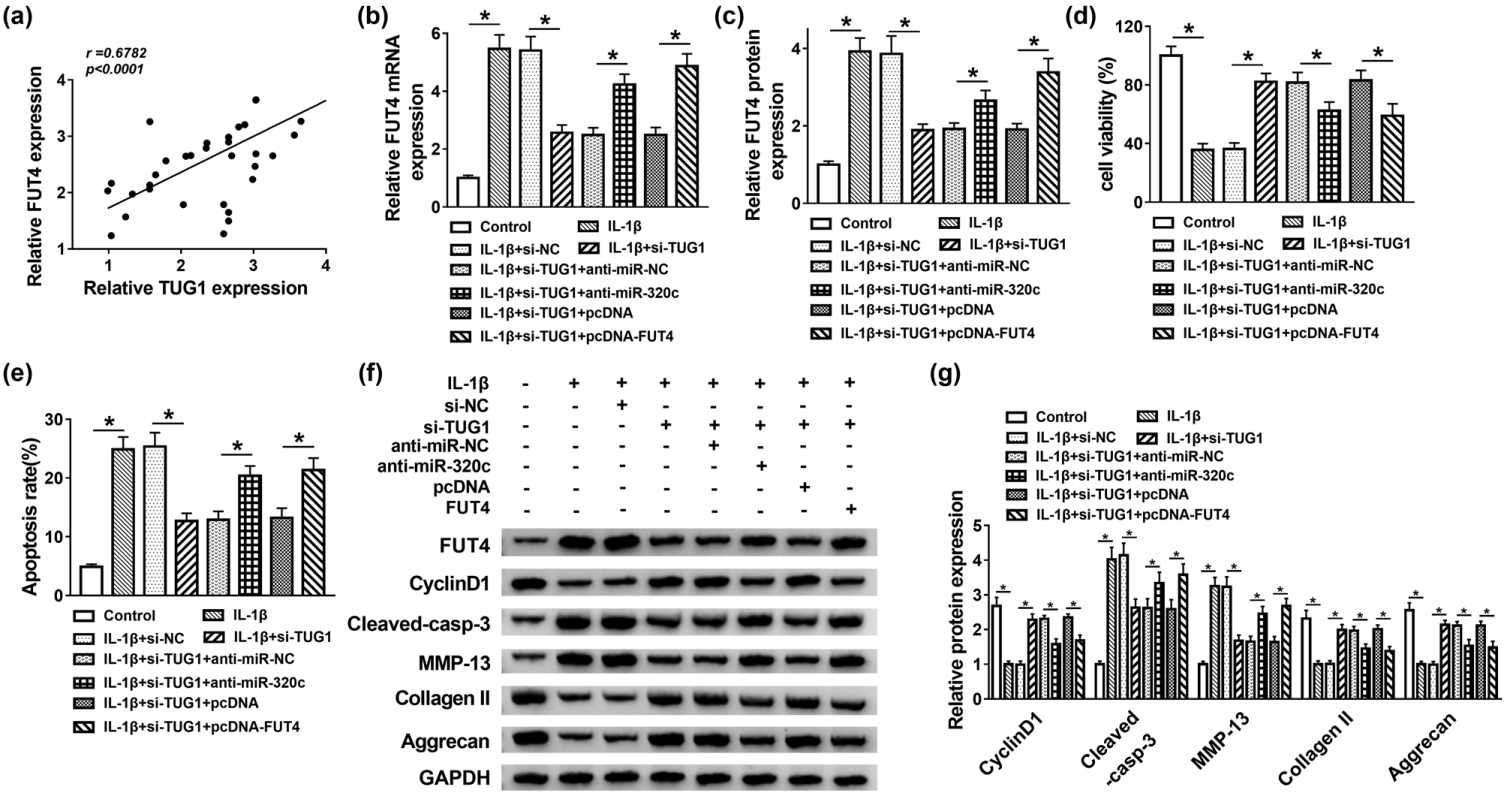
Effects of TUG1 silencing on cell proliferation, apoptosis and ECM degradation were abrogated by miR-320c inhibitor or FUT4 overexpression in IL-1β-stimulated C28/I2 cells. (a) The association between TUG1 and FUT4 was analyzed via Spearman’s correlation analysis. (b–g) si-NC, si-TUG1, si-TUG1 + anti-miR-NC, si-TUG1 + anti-miR-320c, si-TUG1 + pcDNA or si-TUG1 + pcDNA-FUT4 was introduced into IL-1β-induced C28/I2 cells. (b and c) The mRNA level and protein expression were measured by qRT-PCR and Western blot assays, respectively. (d) MTT assay was performed to estimate cell viability *in vitro*. (e) The apoptotic rate was measured by flow cytometry. (f and g) The relative proteins of cyclin D1, cleaved-casp-3, MMP-13, collage II and Aggrecan were determined using Western blot. *P < 0.05.

First, the correlation between FUT4 and TUG1 was analyzed and the results showed that FUT4 expression was positively related to the TUG1 level (Figure 6a). Then the molecular mechanism between TUG1 and miR-320c or FUT4 was explored, and si-NC, si-TUG1, si-TUG1 + anti-miR-NC, si-TUG1 + anti-miR-320c, si-TUG1 + pcDNA or si-TUG1 + pcDNA-FUT4 was transfected into IL-1β-treated C28/I2 cells. The FUT4 mRNA and protein expression were restrained by TUG1 deletion, which was recovered by miR-320c inhibitor or FUT4 overexpression (Figure 6b and c). Moreover, the acceleration effect of TUG1 silencing on cell proliferation in IL-1β-treated C28/I2 cells was blocked by miR-320c inhibitor or FUT4 upregulation (Figure 6d). The inhibitory effect of si-TUG1 on cell apoptosis was abrogated by miR-320c inhibitor or overexpression of FUT4 in C28/I2 cells under IL-1β-treated condition (Figure 6e). Furthermore, the expression of cyclin D1 and cleaved-casp-3 also verified the previously obtained results regarding cell proliferation and apoptosis. Besides, ECM-relative proteins of MMP-13, collage II and Aggrecan were also measured by Western blot assay, and the results displayed that the repression impact of TUG1 deletion on degradation of ECM was overturned by miR-320c inhibitor or FUT4 upregulation in C28/I2 cells with IL-1β stimulation (Figure 6f and g). In short, the influence of TUG1 knockdown on cell proliferation, apoptosis and degradation of ECM was abolished by miR-320c inhibitor or FUT4 overexpression in IL-1β-induced C28/I2 cells.

## 4 Discussion

OA with high incidence severely impacts the life quality of patients [19], while the etiology and pathogenesis of OA remain largely unknown. IL-1β is one of the most vital inflammatory factors in the early stage of OA, which is produced in scathing and degenerated joints. IL-1β was applied to induce OA cell model according to the previous study [20,21]. Recently, multiple research demonstrated that lncRNAs play vital roles in the development of OA [22]. For example, lncRNA PCGEM1, a cartilage injury-related lncRNA, could modify cartilage injury and degradation [23]. lncRNA UFC1 improved the capacity of chondrocyte proliferation via interacting with miR-34a in OA [24]. Before our study, only a single report provided an insight on the relation between TUG1 and OA, suggesting that TUG1 mediated OA progression via targeting miR-195/MMP-13 axis [12]. MMP-13, which was produced from bone and joint, was tightly associated with ECM degradation. Nevertheless, upregulation of MMP-13 could cause degradation of ECM under pathological conditions [25,26]. Moreover, Duan et al. reported that MMP-13, collagen II and Aggrecan expression correlated with ECM degradation [20]. In this study, TUG1 overexpressed in OA cartilage tissue separated OA chondrocytes and IL-1β-treated C28/I2 cells. Moreover, TUG1 silencing dramatically promoted cell proliferation and suppressed apoptosis in IL-1β-treated C28/I2 cells. Besides, MMP-13, collagen II and Aggrecan as the markers for ECM degradation were measured in IL-1β-treated C28/I2 cells. Our data indicated that TUG1 inhibited ECM degradation in IL-1β-treated C28/I2 cells. These findings meant that TUG1 plays an important role in OA progression.

The miRNAs were proved to be related to the progression and pathogenesis of articular cartilage [27,28,29]. Our data indicated that miR-320c may be one of the downstream genes of TUG1. A previous research reported that miR-320 modified MMP-13 expression in IL-1β-stimulated chondrocyte responses [30]. In the present study, the data showed that lower miR-320c level was observed in OA patients and IL-1β-treated C28/I2 cells, which was in accordance with the previous studies [30,31]. Interestingly, the effects of TUG1 knockdown on cell proliferation, apoptosis and degradation of ECM were abolished by miR-320c inhibitor, suggesting that TUG1 exerted functions in IL-1β-treated C28/I2 cells by sponging miR-320c.

As we all know, miRNAs exerted their role via directly regulating the target gene expression, which binds to the 3′-UTR [32]. Owing to the results of bioinformatics tools, subsequent assays of dual-luciferase reporter and RIP were performed to confirm the interaction between miR-320c and FUT4, and all results demonstrated that FUT4 was a target gene of miR-320c. FUT4 was a member of FUT family, which contributed to biological processes, including tissue development, inflammatory response and cancer metastasis [33,34]. A previous study suggested that FUT4 targeted by miRNA mediates OA progression [35]. Hence, we presented a hypothesis that FUT4 targeted by miR-320c participated in the progression of OA. As expected, upregulation of FUT4 relieved the effects of miR-320c on cell proliferation, apoptosis and degradation of ECM in IL-1β-induced C28/I2 cells. Moreover, our results indicated that TUG1 inhibited the expression of FUT4 by targeting miR-320c. Besides, the effects of TUG1 deletion on the proliferation, apoptosis and ECM degradation were abolished by miR-320c inhibitor or FUT4 overexpression in IL-1β-induced C28/I2 cells, suggesting that TUG1 was involved in the progression of OA through regulating FUT4 by sponging miR-320c.

## 5 Conclusion

In summary, the levels of TUG1 and FUT4 were obviously upregulated, while miR-320c level was significantly decreased in OA and separated chondrocytes as well as IL-1β-stimulated C28/I2 cells. Mechanically, miR-320c was targeted by TUG1 while directly targeting FUT4. Functionally, TUG1 silencing promoted cell proliferation and suppressed cell apoptosis and ECM degradation in IL-1β-induced C28/I2 cells. Moreover, FUT4 overexpression reversed miR-320c effect on cell proliferation, apoptosis and ECM degradation in IL-1β-treated C28/I2 cells. Besides, these effects of TUG1 knockdown on IL-1β-treated C28/I2 cells were rescued by miR-320c inhibitor or FUT4 overexpression. More importantly, TUG1 targeted miR-320c to regulate FUT4 expression in IL-1β-treated C28/I2 cells. Taken together, TUG1 modified cell proliferation, apoptosis and ECM degradation via the miR-320c/FUT4 axis in IL-1β-treated C28/I2 cells, providing a novel molecular target in treating OA.
